# Adult Wilms Tumor With Inferior Vena Cava Thrombus on an Incomplete Duplex Collecting System Ureter Fissus Proximalis Managed at a Tertiary Hospital in Tanzania: A Case Report and Literature Review

**DOI:** 10.1002/ccr3.70136

**Published:** 2025-01-22

**Authors:** Amini Mitamo Alexandre, Joseph Martin Lori, Gabriel F. Mtaturu, Victor Patrick Sensa, Sylvia Bedas Nsato, Mariam Athumani Mbezi, Obadia V. Nyongole, Charles A. Mkony

**Affiliations:** ^1^ Department of Surgery Muhimbili University of Health and Allied Sciences Dar es Salaam Tanzania; ^2^ Department of Urology Muhimbili National Hospital Dar es Salaam Tanzania; ^3^ Department of Radiology Muhimbili National Hospital Dar es Salaam Tanzania; ^4^ Department of Pathology Muhimbili National Hospital Dar es Salaam Tanzania

**Keywords:** adjuvant chemotherapy, adult Wilms' tumor, inferior vena cava thrombus, radical nephrectomy, ureter fissus proximalis

## Abstract

Wilms' tumor (WT), also known as nephroblastoma, is a malignant embryonal kidney tumor composed of embryonic cells and is the most prevalent tumor among children, but isolated cases occur infrequently in the adult population. Adult WT is defined according to the criteria of Kilton, Matthews, and Cohen, which comprise age above 15 years and histological patterns characteristic of WT. We report a case of an adult WT with venous thrombus on an incomplete duplex collecting system. To the best of our knowledge, this is the first case of adult WT with such a presentation. A 28‐year‐old female patient presented to our department with a 4‐month history of right flank pain and flank mass and was diagnosed by abdominal contrasted CT to have a right renal tumor with tumor thrombi in the renal vein and the inferior vena cava. The CT scan also revealed a bilateral duplex collecting system with a partial (ureter fissus proximalis) on the tumor side and a complete duplex system on the contralateral side. Right radical nephrectomy with complete tumor thrombectomy via venacavotomy was performed successfully. Histopathological examination of the specimen revealed a triphasic nephroblastoma with immunohistochemistry confirmation. Postoperatively, adjuvant chemotherapy was initiated. The increasing incidence of non‐syndromic WT cases associated with duplex collecting systems suggests a potential shared pathogenesis, necessitating further research.

## Graphical abstract

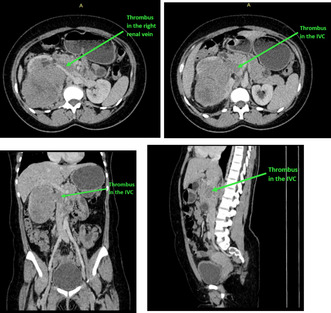

**Summary**

- Adult Wilms' tumor (WT) is defined by the presence of histological patterns of WT in a patient older than 15 years. It is a rare and challenging diagnosis, often mistaken for renal cell carcinoma due to its infrequent occurrence and lack of distinct radiological features. Unlike pediatric cases, adults typically present with symptoms such as flank pain and hematuria rather than a painless abdominal mass.
- Histologically, WTs typically exhibit triphasic pattern composed of blastemal, stromal, and epithelial tubule components. Biphasic or monophasic patterns are rare.
- The presence of a duplex collecting system, though rare, has been associated with WT, sometimes within the context of syndromes like WAGR. Isolated cases have also been reported.
- Venous involvement, including inferior vena cava thrombus, is well documented in pediatric cases but less so in adults.
- There are no standardized treatment guidelines for adult WT, due to rarity of WT in adults, and managing this condition has been challenging. Existing protocols have been adapted from the multimodal approach used in pediatric patients, which includes surgery, chemotherapy, and radiation therapy.

**Abbreviations**
3DVMs: Three‐dimensional virtual models
CECT: contrast enhanced computed tomography
CGO: Children's Oncology Group
eGFR: estimated glomerular filtration rate
IHC‐WT1: immunohistochemistry‐Wilms tumor gene 1
IVC: inferior vena cava
PAX‐8: paired‐box gene 8
SIOP: Société Internationale D'oncologie Pédiatrique
WAGR: Wilms tumor, Aniridia, Genitourinary anomalies, and Range of developmental delays
WT: Wilms tumor

## 1 Introduction

Wilms' tumor (WT), also known as nephroblastoma, is a malignant embryonal kidney tumor composed of embryonic cells that are typically prevalent among children under the age of five [1].

While WT is recognized as the most prevalent renal neoplasm in children, it infrequently occurs in adults, with an incidence of 0.2 per million (equivalent to 70 newly diagnosed cases annually in Europe) [2].

The diagnosis of adult WT is made according to Kilton, Matthews, and Cohen, who suggested specific criteria. These criteria include (a) confirmation that the tumor is a primary renal neoplasm; (b) the identification of a round cell or primitive blastemic spindle component; (c) the presence of abortive or embryonal tubules or glomerular structures; (d) the absence of typical areas indicative of renal cell carcinoma; (e) histological confirmation through visual evidence; and (f) age greater than 15 years [3].

In the English literature, about 300 cases of adult WT have been documented [2, 4], with only a limited number of cases detailing the involvement of the inferior vena cava (IVC) [2].

Most cases of WT are sporadic with genetic predispositions being found in 10%–15%, most of them involving overgrowth or a genitourinary anomaly. Although very rare, WT is associated with genitourinary anomalies [5].

In this report, we present a rare case of an adult patient with a WT on a partial duplex collecting system with a tumor thrombus in the right renal vein and IVC.

## 2 Case History/ Examination

A 28‐year‐old female with right flank pain and a mass for 4 months was admitted to our department. No hematuria or any systemic symptoms were reported. The patient's medical history revealed no other notable events. There was no family history of cancer or other medical issues. The individual was a nonsmoker, had no allergies, and was not on any regular medications.

## 3 Investigations and Treatment

**FIGURE 1:**
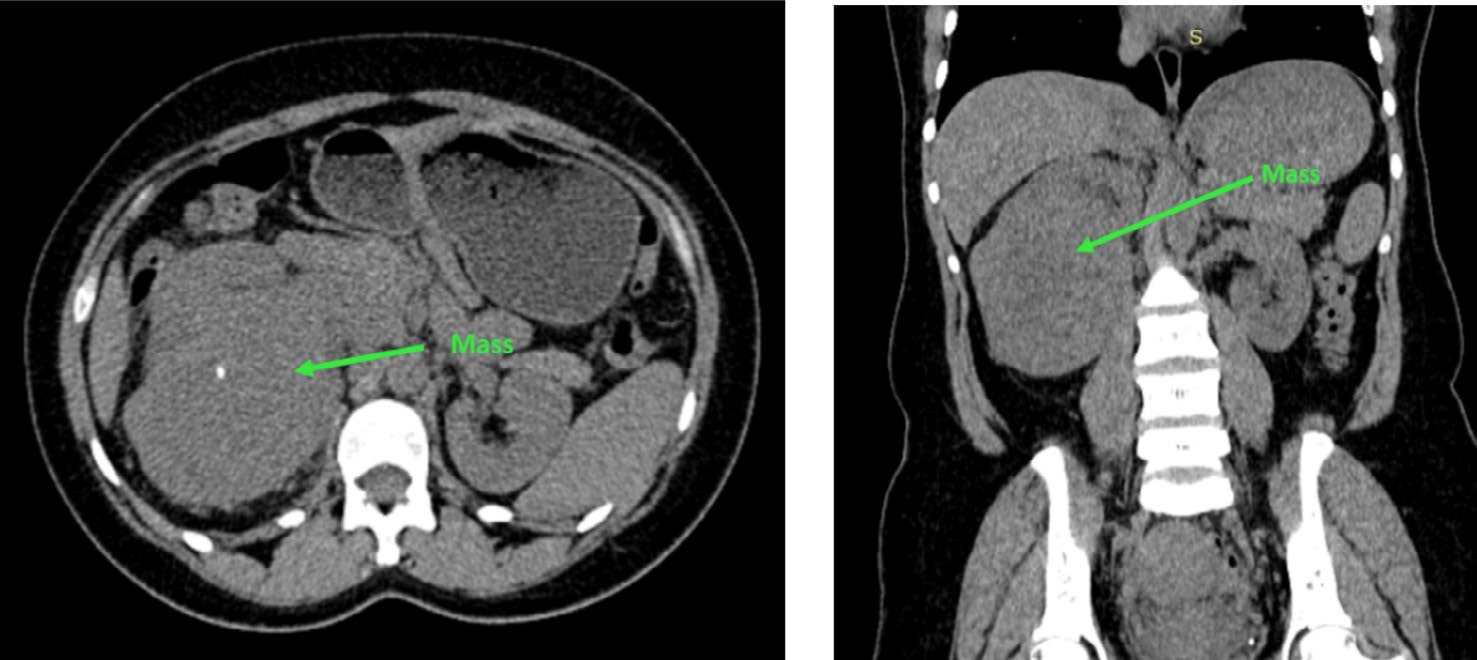
Pre‐contrasted axial and coronal CT scans of the abdomen showing a soft tissue mass in the right kidney with a calcified foci.

**FIGURE 2:**
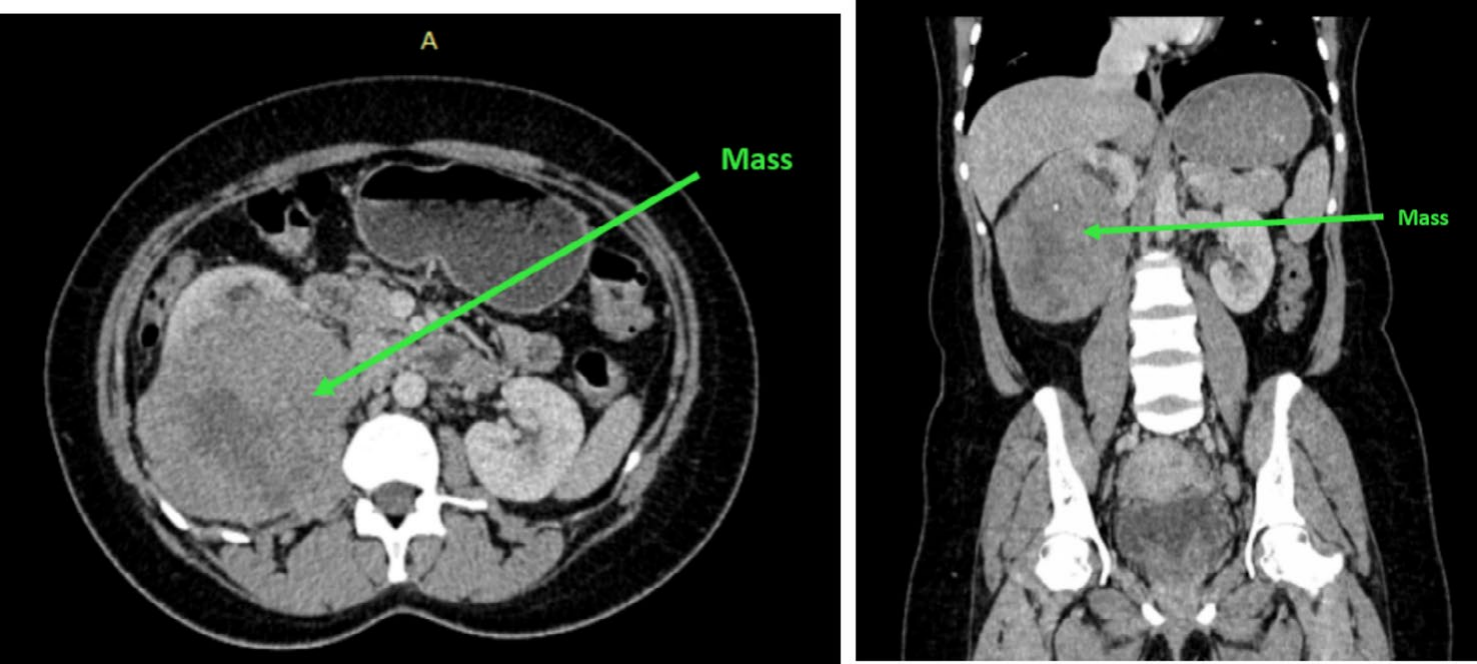
Contrasted axial and coronal CT scan images of the abdomen showing a large heterogeneous mass arising from the right kidney's lower pole and displacing the remaining renal parenchyma anteromedially. The mass contains mixed solid and cystic areas measuring about 10.11 × 9.62 cm. The mass shows a normal nephrogram and excretion.

**FIGURE 3:**
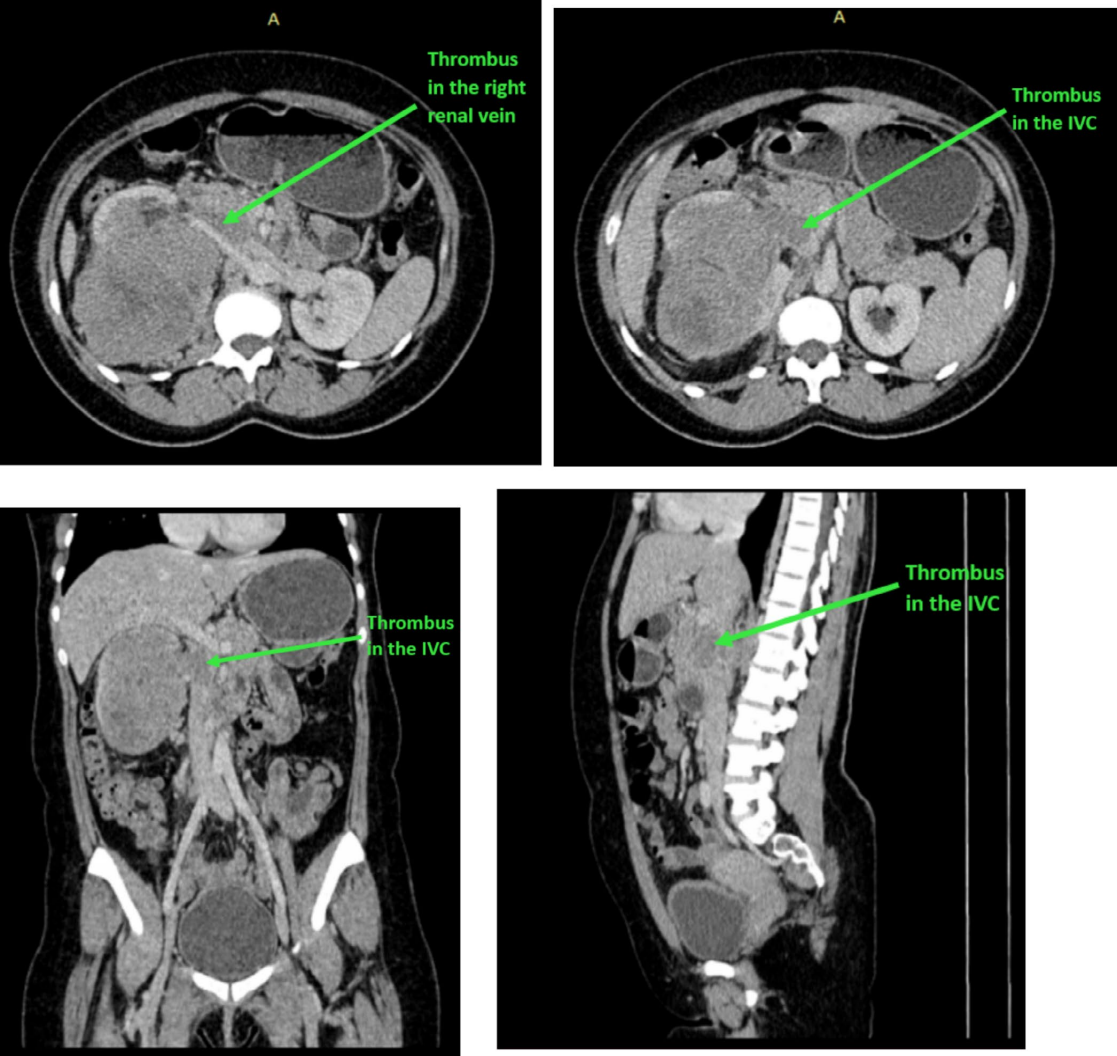
Contrasted axial, coronal, and sagittal CT scan of the abdomen showing extensive tumor thrombus filling the right renal vein and the IVC.

**FIGURE 4:**
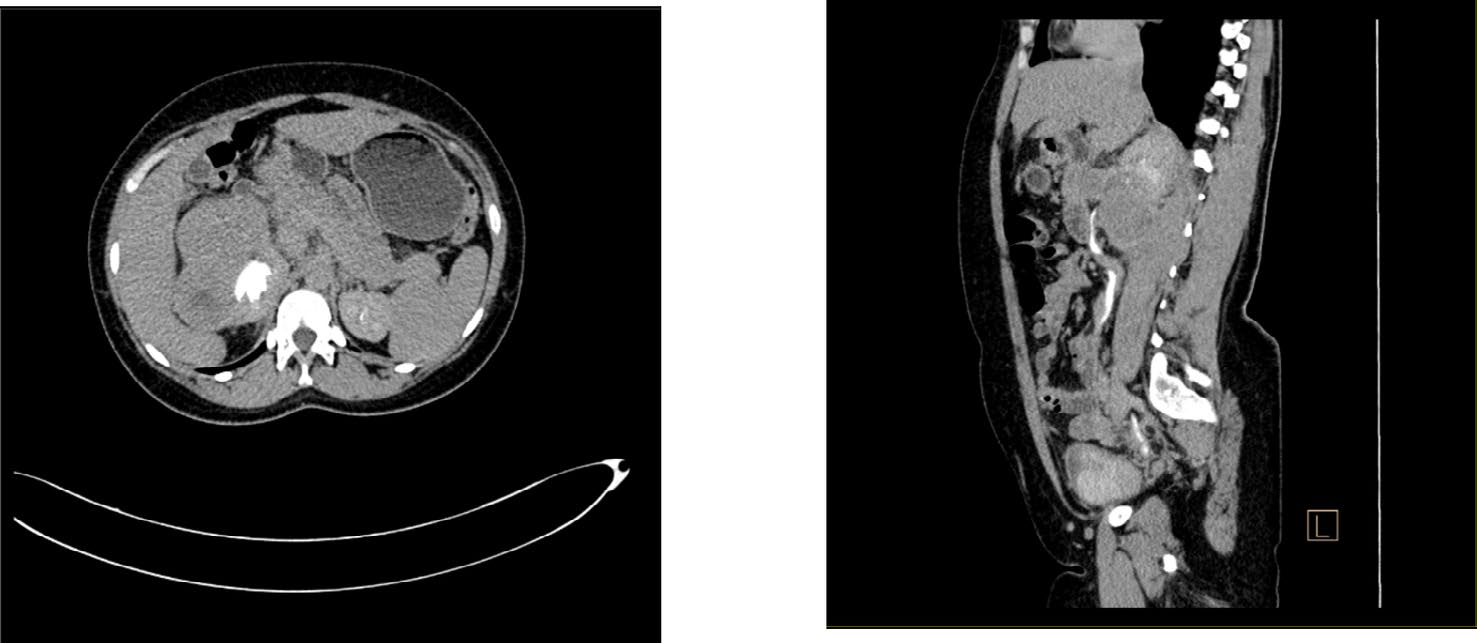
Contrasted axial and coronal delayed CT images of the abdomen showing normal excretion.

**FIGURE 5:**
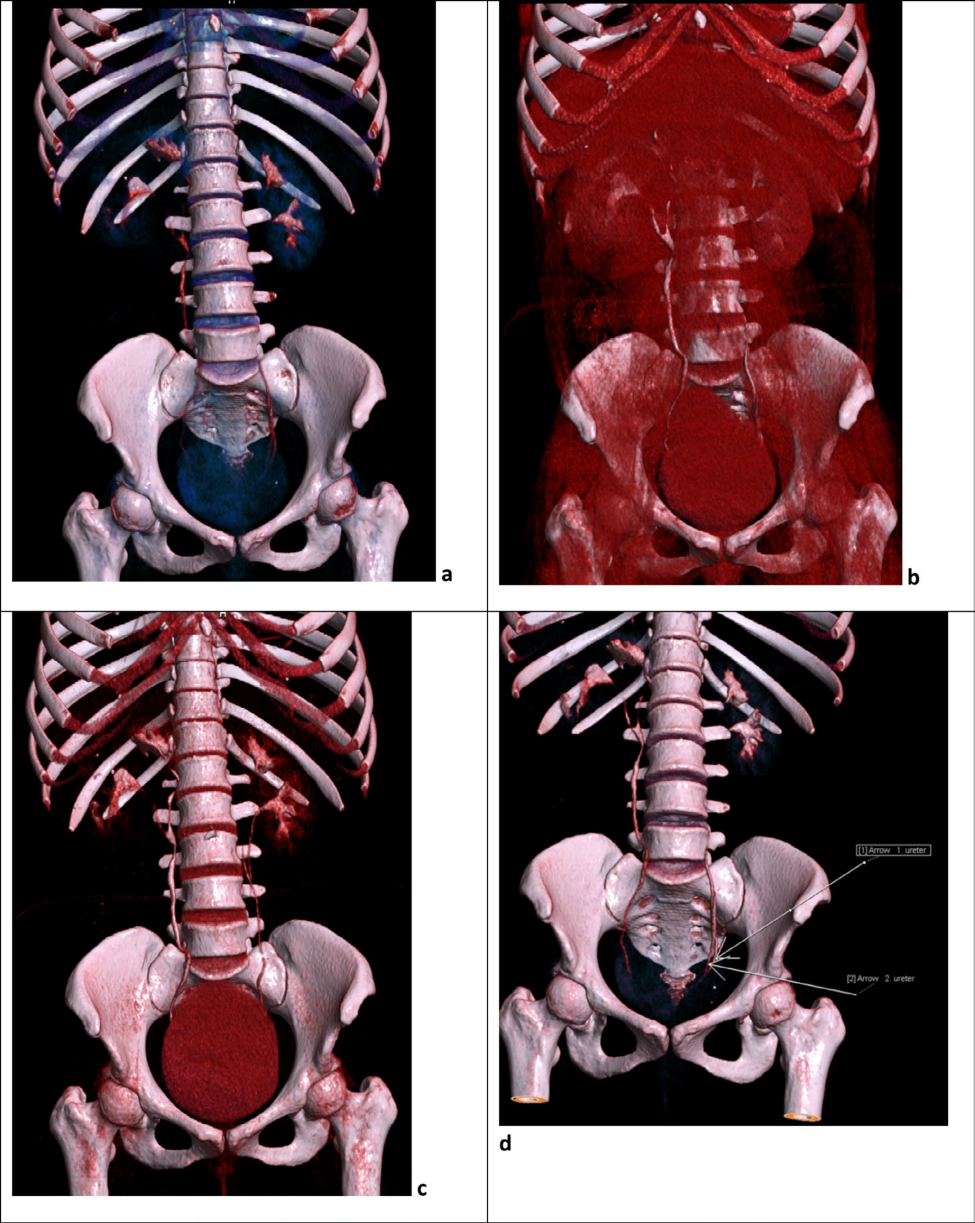
CT volumetric images of the abdomen showing the right duplex collecting system draining into a single ureter and a duplex collecting system with a double ureter (complete duplication with two ureters that drain separately into the bladder) in the left kidney.

The patient was attended to at our outpatient clinic, where an abdominal ultrasound was performed, which revealed a large heterogeneous mass at the lower pole of the right kidney. A contrast‐enhanced abdominal computed tomography (CECT) was recommended for further evaluation of the mass. The CECT scan showed a heterogeneously enhancing solid renal mass with areas of necrosis in its lower pole measuring 10.64 cm x 10.40 cm, associated with extensive tumor thrombus filling the right renal vein and IVC (level II). The scan also revealed a right duplex collecting system draining into a single ureter and a normal left kidney with a complete duplex collecting system (Figures 1, 2, 3, 4, 5). Hematological investigations revealed a hemoglobin level of 10.40 g/dL. Urine analysis revealed no hematuria or proteinuria, and baseline biochemical tests were within normal ranges. The eGFR was 71.192 mL/min/1.73 m^2^, and the chest X‐ray was unremarkable. An echocardiogram revealed a normal study, with an ejection fraction of 65%.

The provisional diagnosis of primary renal cell carcinoma stage III was made based on clinical and investigation findings. An elective surgery was scheduled. Open radical nephrectomy with complete vena cava tumor thrombus extraction via wide venacavotomy was performed. There were no residual tumor masses left. The procedure lasted for 3 h, and the estimated blood loss was around 800 ml which necessitated an intraoperative blood transfusion of 4 units of packed red blood cells. The patient had an uneventful postoperative course and was discharged home on the fifth day postoperatively.

**FIGURE 6:**
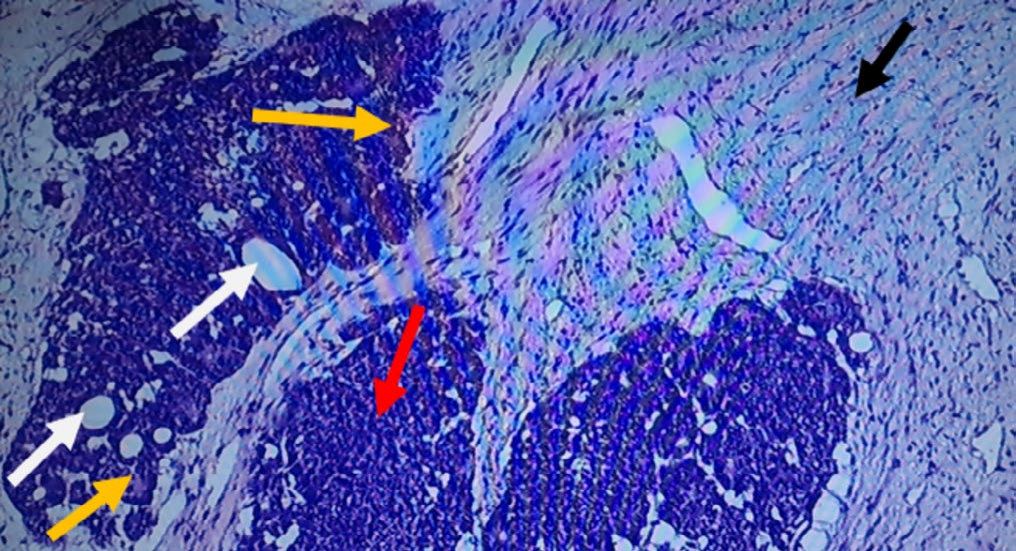
Triphasic tumor composed of an epithelial component corresponding to well‐formed tubules (white arrows) and rosettes (yellow arrows), blastema component (red arrow), and mesenchymal component characterized by stromal undifferentiated cells (black arrow).

**FIGURE 7:**
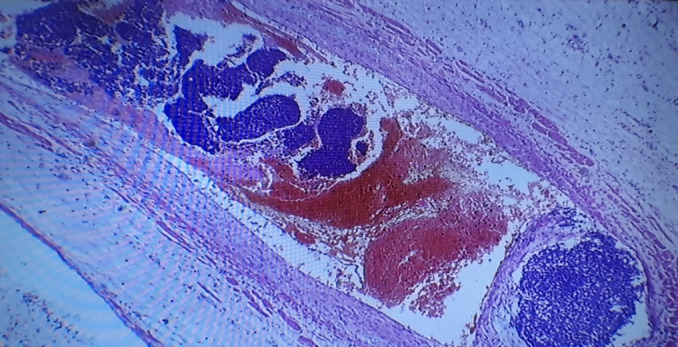
Tumor thrombi in the IVC.

**FIGURE 8:**
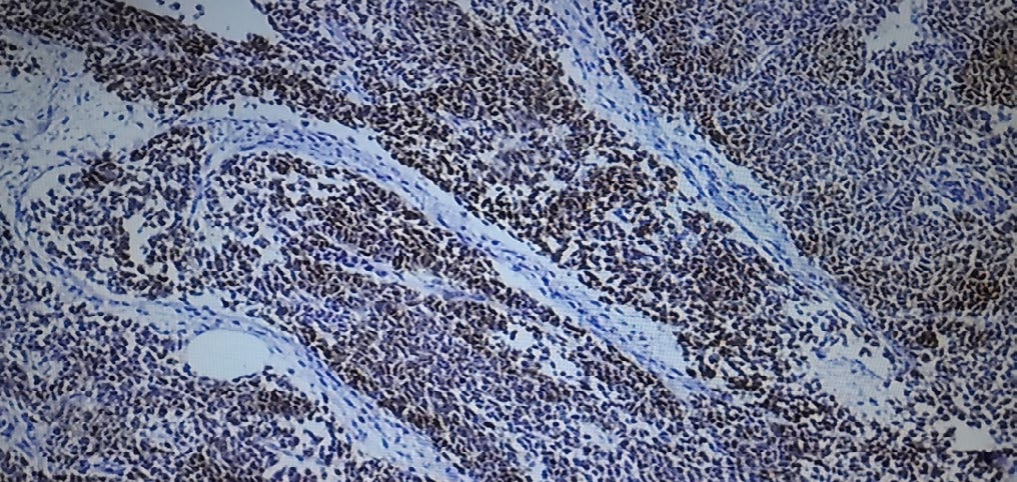
WT1.

**FIGURE 9:**
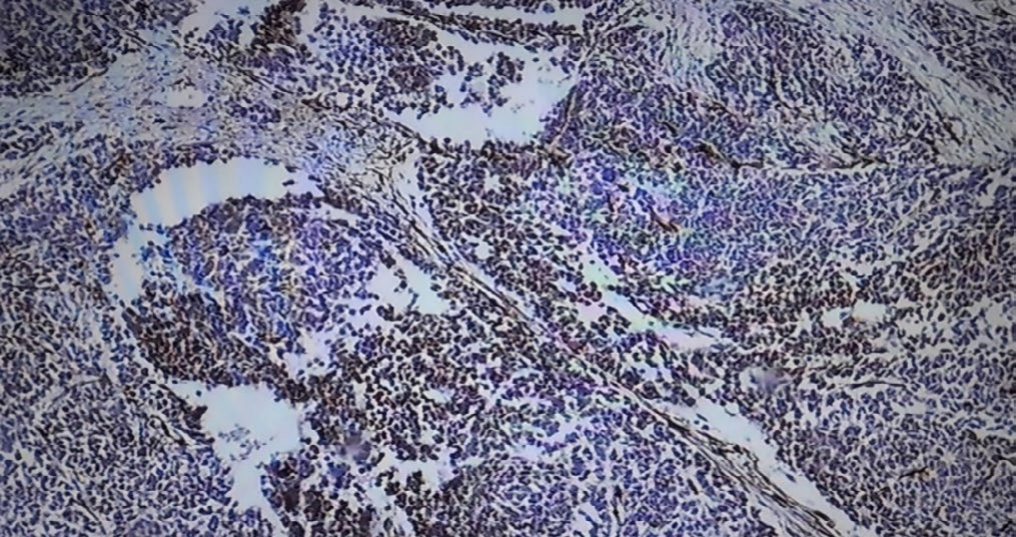
PAX‐8.

The specimen was submitted for histology, and the following macroscopic and microscopic findings were noted: Grossly, it presented as a specimen with a huge tan‐white mass occupying the entire kidney, leading to a disfigurement of its morphology and orientation. The tumor measured 15 × 9 × 9 cm and was soft to firm with an intact capsule. Microscopically, there was the presence of large sheets and nests of blastema cells, well‐formed tubules, primitive tubules (rosettes), and an abundant loose stroma of undifferentiated cells, consistent with WT (Figure 6). Vascular tumor thrombi were also observed (Figure 7). Immunohistochemistry showed nuclear positivity with WT1 and PAX8 in the epithelial component (Figures 8 and 9). The final COG staging was stage II WT.

## 4 Outcome and Follow‐Up

The patient was initiated on chemotherapy 2 weeks postoperatively. This was made up of ifosfamide 8400 mg, etoposide 170 mg, and mesna 850 mg. A total of six cycles was administered.

At the follow‐up visit after 4 months, the patient demonstrated excellent tolerance to the chemotherapy regimen, exhibiting no symptoms and a normal clinical assessment.

## 5 Discussion

WT, or nephroblastoma, constitutes a distinct form of renal cancer primarily observed in pediatric patients [1, 2, 5, 6, 7, 8, 9, 10, 11, 12, 13]. The diagnosis of adult WT is made according to Kilton, Matthews, and Cohen criteria [3], and our patient met all the specified diagnostic criteria. We present a case of a primary renal mass with triphasic components that do not exhibit areas indicative of renal cell carcinoma on histology in a 28‐year‐old female patient.

Although extensively studied in children, the occurrence of WT in adults presents diagnostic and, consequently, therapeutic challenges. The clinical presentation of nephroblastoma in adults differs from that in children. In adults, the initial symptoms are pain and hematuria, whereas children present with a painless, palpable abdominal mass that rapidly increases in size [7]. Our patient presented with a long‐standing history of flank pain and a flank mass with no history of hematuria.

Given their infrequent occurrence in adult populations, constituting only 0.5% of all adult renal neoplasms, and the absence of distinct radiological features, most adult WTs are often misdiagnosed clinically as renal cell carcinoma, the most common adult renal malignancy, and are typically diagnosed by histology of the nephrectomy specimen [4]. This was the scenario for our patient, which reflects this common diagnostic challenge.

Approximately 90% of WTs present with blastemal, stromal, and epithelial tubule histologic components. The relative proportions of these elements can vary. In addition to these primary components, some rare tumors may exhibit atypical components such as mucinous or squamous epithelium, skeletal muscle, cartilage, osteoid, or fat [11]. The histology findings have been used for risk stratification, using the Children's Oncology Group (COG) Renal Tumor Group Histology Classification when neoadjuvant chemotherapy was not initiated, or the International Society of Paediatric Oncology (SIOP) Histology classification when the tumor was treated with neoadjuvant chemotherapy. Tumors not treated with neoadjuvant chemotherapy are of favorable histology when they exhibit blastemal, epithelial, and stromal cells with no anaplasia. They are unfavorable if they present with atypical multipolar mitotic figures, hyperchromic nuclei, and significant nuclear enlargement [14].

In our patient, the histology analysis revealed a favorable histology made of a triphasic tumor, and immunohistochemistry showed positivity for WT1 and PAX8.

Venous involvement in childhood Wilms tumor is common and is observed in up to 45% of cases. Tumor thrombosis in the IVC is noted in 4%–10% of patients, with 1%–3% displaying involvement in the right atrium. Although these statistics align with the IVC tumor extension rates observed in adult patients with renal cell carcinoma (RCC), the frequency of venous involvement in adult WTs remains unknown. At present, there is limited literature on adult WT with IVC thrombus on an incomplete duplex collecting system, with only a few case reports available [2]. The patient we present had a tumor thrombus filling the renal vein and the IVC below the insertion of the hepatic vein. This corresponds to Level II according to the Mayo Clinic Classification as described by Hatakeyama et al. [15] This classification is summarized in Table 1.

**TABLE 1:** Classification of tumor thrombus level according to the Mayo staging system [15].

Level 0	Thrombus extending to the renal vein.
Level I	Thrombus extending into the IVC up to 2 cm above the renal vein.
Level II	Thrombus extending into the IVC to more than 2 cm above the renal vein but not reaching the level of the hepatic vein.
Level III	Thrombus extending into the IVC to above the hepatic vein but below the diaphragm.
Level IV	Thrombus extending into the supra‐diaphragmatic IVC or right atrium.

The occurrence of ureteral duplication in kidney tumors is infrequent, and all cases of such ureteral duplication are found incidentally when investigating renal tumors. This particular association has been reported with various histological types, across s different age groups, and has been found as part of syndromes [8, 16]. A duplex system refers to a renal unit in which the kidney has two pelvicalyceal systems, consisting of partial or complete duplication of the ureters [16, 17]. In the case of a complete ureteral duplication, each renal moiety has a distinct whole‐length ureter. Incomplete duplex systems or ureter fissus are those in which these distinct ureters fuse before the ureterovesical junction. Further subdivision of incomplete duplex systems includes the bifid pelvis (or ureter fissus proximalis), where fusion occurs in the proximal ureters, and the bifid ureter (or ureter fissus distalis), where fusion occurs at a more distal point [17, 18]. The association between WT and the duplex collecting system has been observed in WAGR syndrome, characterized by WT, Aniridia, Genitourinary anomalies, and a range of developmental delays [19, 20]. However, this association has also been noted independently, outside of the context of WAGR Syndrome. In the literature, the available data on cases of WT and duplex collecting systems outside of WAGR syndrome are summarized in Table 2.

**TABLE 2:** Isolated Wilms' tumor on a duplex collecting system: Literature review.

Series	Age	Sex	Type	Side	Moiety involved by the tumor	Year
Dietz, Wahlen, and Kastert [21]	8 months	Female	Complete	Left	Upper	1978
Zirn, Wittmann, and Gessler [22]	9 years	Female	Complete	Right	—	2005
Kajbafzadeh et al. [8]	4 years	Male	Complete	Left	—	2013
Karnak et al. [13]	10 years	Female	Complete	Right	Upper	2017
Zhao et al. [9]	5 years	Female	Partial (ureter fissus)	Left	Lower	2021
Wu et al. [10]	6 years	Female	Complete	Right	Lower	2023

The current pathogenesis of this isolated association remains unclear, leading previous researchers to consider whether there is a shared pathogenesis or if this concurrent occurrence is simply coincidental [8]. To the best of our knowledge, this is the first case of adult WT with an IVC tumor thrombus in a patient who presented with a bilateral duplex kidney and a ureter fissus proximalis type on the affected side.

Due to the rarity of adult WT, standardized therapy is lacking, which poses significant challenges for urologists and oncologists in managing adult patients. Protocols have been adapted from pediatric treatments, with case series demonstrating improved survival rates when these pediatric protocols are applied [14, 23].

The current management plan of WT in adults is based on consensus between renal tumor committees of the International Society of Pediatric Oncology (SIOP) and Children's Oncology Group (COG). The consensus recommends that for nonmetastatic, operable tumors, immediate nephrectomy should be performed, followed by proposed postoperative protocols (chemotherapy with or without flank radiation) depending on tumor stage and histological risk classification [14]. Experts also recommend initiating chemotherapy and radiotherapy if necessary, as early as by Day 14–30 postnephrectomy since delayed initiation of adjuvant therapy has been found to be associated with a significant decrease in the 5‐year event‐free survival rate [14, 23]. Our patient received adjuvant chemotherapy based on the ifosfomide–etoposide–mesna regimen, and this was started from Day 14 post‐surgery. Despite its usual indication for unresponsive or relapsed WT, this regimen was selected for our patient in place of vincristine, dactinomycin, and doxorubicin [24] due to the limited availability of these latter agents in our setting and considerations regarding patient affordability.

In recent years, renal tumor surgery has advanced with the use of minimally invasive techniques supported by three‐dimensional virtual models (3DVMs). These models offer significant intraoperative benefits, including highly detailed anatomical representations and guidance throughout the procedure. The utility of 3DVMs has been assessed in the robotic management of renal tumors with vena cava thrombus, where the extent of the thrombus was accurately identified [25]. In our case, an open approach was performed because these technologies are not available at our facility.

Neoadjuvant chemotherapy is considered in adults with metastatic disease or surgically inoperable tumors [14]. This is in contrast with the SIOP protocol in pediatric patients, which advocates in this population a standard chemotherapy protocol before surgery even out of the metastatic stage because it personalizes in vivo assessment of histological response to chemotherapy, including the identification of a “high‐risk” category of blastemal‐type WT. Neoadjuvant chemotherapy has also been found to lead to a reduction in tumor size and the formation of a fibrous pseudo‐capsule that facilitates surgical removal, decreases the risks of rupture and spillage during surgery, and decreases the risk of intraoperative hemorrhage [12].

## 6 Conclusion

In adults, WT is often initially misdiagnosed as renal cell carcinoma due to the absence of distinct radiological findings. Histological examination is crucial for an accurate diagnosis and staging. Adjuvant chemotherapy remains an option when the histological diagnosis is made postoperatively. This case underscores the importance of considering WT in the differential diagnosis of renal masses in adults and highlights the need for further research to understand the unique aspects of this disease in adult patients, including its association with congenital anomalies and optimal management strategies to improve patient outcomes. The increasing number of documented cases of non‐syndromic WTs on duplex collecting systems suggests the possibility of a shared common pathogenesis rather than a coincidence—a hypothesis to be clarified through future studies.

## Author Contributions

**Amini Mitamo Alexandre:** conceptualization, writing – original draft. **Joseph Martin Lori:** writing – original draft. **Gabriel F. Mtaturu:** supervision, writing – review and editing. **Sylvia Bedas Nsato:** investigation. **Mariam Athumani Mbezi:** investigation. **Victor Patrick Sensa:** supervision, writing – review and editing. **Obadia V. Nyongole:** supervision, writing – review and editing. **Charles A. Mkony:** supervision, writing – review and editing.

## Ethics Statement

The authors have nothing to report.

## Consent

Written informed consent was obtained from the patient to publish this report in accordance with the journal's patient consent policy.

## Conflicts of Interest

The authors declare no conflicts of interest.

## Data Availability

The authors have nothing to report.
